# Automotive suspension component behaviors driven on flat and rough road surfaces

**DOI:** 10.1016/j.heliyon.2021.e07528

**Published:** 2021-07-09

**Authors:** T.E. Putra, Husaini, M. Ikbal

**Affiliations:** Department of Mechanical Engineering, Faculty of Engineering, Universitas Syiah Kuala, Darussalam, 23111, Banda Aceh, Indonesia

**Keywords:** Fatigue, Strain, Stress, Vibration, Life

## Abstract

The objective of this study is to identify the behavior of the car suspension components subjected to road surface contours. Strain signals were measured by installing a strain gauge at the critical area of the coil spring and lower arm. The car was driven on a flat and rough road surface with speeds of 30–40 km/h and 10–20 km/h, respectively. According to the fatigue life assessments based on the strain-life approach, it was found that when the car was driven on the rough road, the components received higher stresses, contributing to a shorter fatigue life. The fatigue life of the coil spring when being driven on the rough road was 1,248 cycles to failure, which was more than 14 times shorter when being driven on the flat road, with 19,060 cycles to failure. Meanwhile the fatigue life of the lower arm being driven on the rough surface was 3,580 cycles to failure, which was almost 3,328 times shorter when being driven on the flat road, with 11,914,000 cycles to failure. The useful life of the coil spring was more than 625 times lower than the lower arm when driven on the flat road, whereas when driven on the rough road, the useful life of the coil spring was almost 3 times lower than the lower arm. In conclusion, the coil spring will fail more than 2 times faster than the lower arm. This is because the contour of the road surfaces provide a vertical load, directly working the coil spring which reduces the load vertically, while the lower arm functions to hold the load when turning.

## 1 Introduction

Dynamic friction between the car tire and road surface produces vibrations which increase when an uneven road surface is paased at high speeds. A repeated vibration causes significant fatigue damage to all components. Moreover, a fatigue failure refers to the formation and propagation of cracks in engineering structures. It is responsible for approximately 90% of the overall mechanical components' failures [1]. Through fatigue analysis, the damage risk caused by repeated load, and the life cycle of any mechanical component that has to receive or bear the load are estimated. Hence, this helps in decreasing the damage risk to ensure the component satisfies the design targets.

Based on these cases, reducing vibration is immensely beneficial for inhibiting fatigue failure and this function is performed by two vital components, namely coil spring and lower arm. A coil spring in the car holds, decreases and absorbs the cyclic impact and torsional loads [2, 3, 4]. Contrarily, the lower arm connects the car suspension system to its mainframe, which further helps in controlling the forward and reverse movements [5]. Roman et al. [6] analyzed more than 3,000 repaired cars and concluded that the automotive suspension components need to be replaced early, i.e., within five years or after travelling over a distance more than 73,500 km. The data derived from the Ministry of Transport, UK, indicated suspension components had a very high fault rate, i.e., 13.18% of all 24.2 million cars tested [7].

The components' failure was attributed to the residual stresses that negatively affected their durability [8, 9, 10]. Previously, the fatigue life prediction was completed based on strain signals obtained from a coil spring [11, 12, 13, 14, 15] and a lower arm [16, 17] separately. Therefore, this study aims to predict fatigue life in both components simultaneously to examine the reaction of each when being used on various road surfaces. The life of any mechanical component is often affected by the road surfaces which is a factor that also needs to be considered before attempting the prediction.

## 2 Methods and materials

### 2.1 Chemical composition test

**Figure 1:**
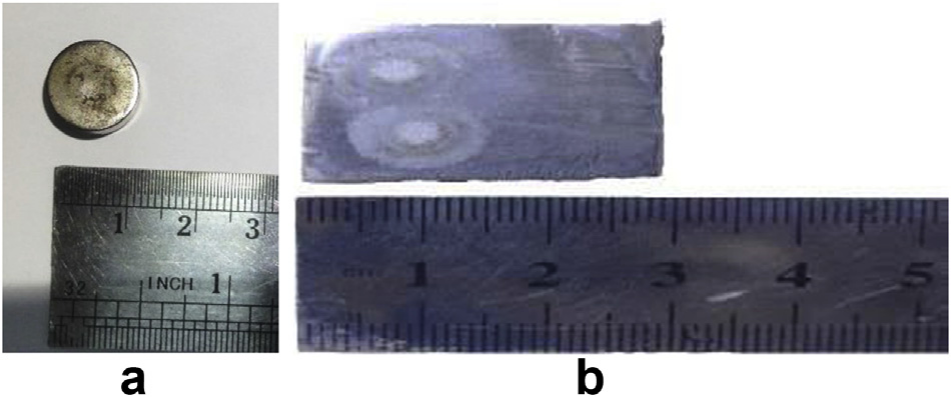
Specimens for the chemical composition tests; (a) coil spring and (b) lower arm.

A chemical composition test was conducted to determine the compositions contained in the coil spring and the lower arm according to ASTM E350-95 [18]. The testing samples (Figure 1) need to be 1–10 cm in size, for easy grinding to ensure the surface is clean of contaminants that can alter the expected results. They were inserted into the PDA 7000 YS (Shimadzu Corp., Japan) and the argon fluidized electrode was subsequently inserted into the samples for ±1 min.

### 2.2 Finite element analysis

**Figure 2:**
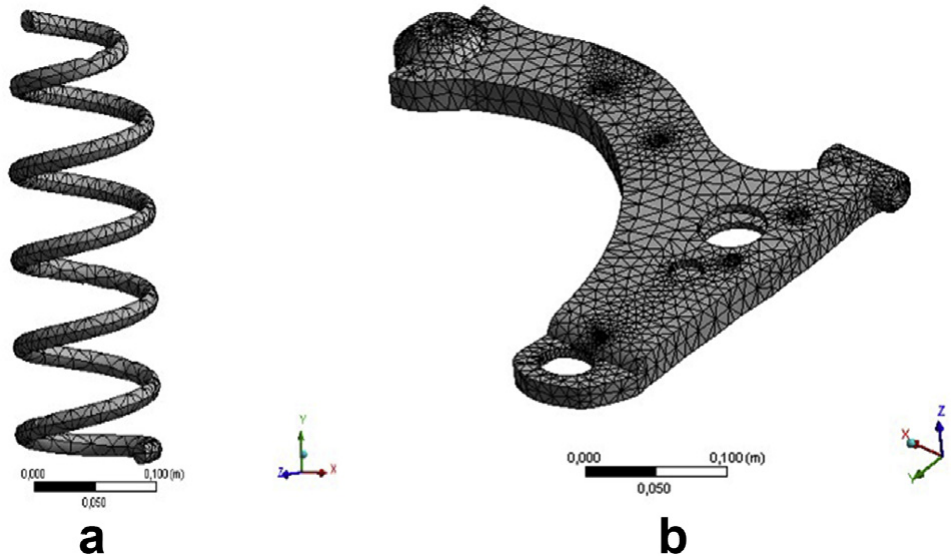
Meshing; (a) coil spring and (b) lower arm.

Design parameters for the coil spring included 285 mm length, 127 mm outer diameter, 13 mm coil diameter, 55 mm distance between coils, and the number of coils which was 6, while those for the lower arm included 411.73 mm length, 332.92 mm height, and 52.02 mm thickness. The meshing utilized for the simulation purpose to ensure a better result is produced was linear tetrahedron [19], as shown in Figure 2. It provided 10,999 nodes and 3,710 elements for the coil spring, and 36,562 nodes and 18,530 elements for the lower arm.

**Figure 3:**
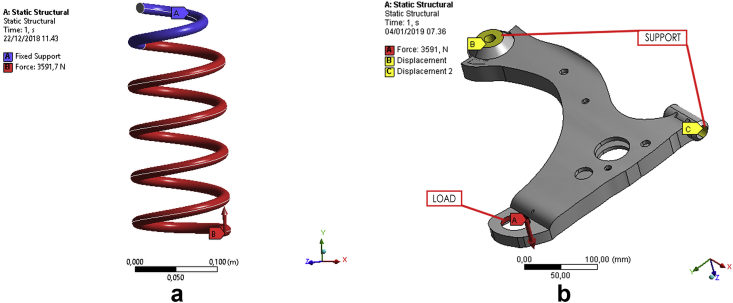
Boundary conditions; (a) coil spring and (b) lower arm.

Figure 3 describes the mechanism used for offering load and support to the suspension components. When the weights of the car and all passengers were 1,045 kg and 420 kg, respectively, the total was divided by four, based on the assumption that the load was evenly distributed on all the wheels such that each has to bear a load of 366.25 kg or 3,591.7 N. This load was subjected at the bottom end of the coil spring (point B), but the upper region offered fixed support (point A). In the lower arm's case, the load was borne by the ball joint (point C), but the bushing sections (points A and B) were offered roller support.

von Mises stress [20] is acquired from the hoop, longitudinal, and radial directions. The yield criterion states that yielding occurs when the equivalent stress $\sigma_{e}$ equals the yield strength $\sigma_{ys}$, as defined by:

(1) $$\sigma_{e}=\frac{1}{\sqrt{2}}{\left[{\left(\sigma_{x}-\sigma_{y}\right)}^{2}+{\left(\sigma_{y}-\sigma_{z}\right)}^{2}+{\left(\sigma_{z}-\sigma_{x}\right)}^{2}+6\left(\tau_{xy}^{2}+\tau_{yz}^{2}+\tau_{zx}^{2}\right)\right]}^{1/2}$$

where $\sigma_{x},\;\sigma_{y},\;\sigma_{z}$ are the normal stresses in the direction of the *x*, *y* and *z*-axes, respectively, while $\tau_{xy},\;\tau_{yz},\;\tau_{zx}$ are the shear stresses.

### 2.3 Strain signal measurement

Strain signal measurements were carried out through 3-mm strain gauge installation on the coil spring and the lower arm, by choosing these locations according to where the highest stress was distributed. Its sampling frequency needs to be greater than 400 Hz to prevent the essential signal components from missing [21]. This indicates a 500 Hz frequency is sufficient for determining and storing the destructive load cycles in both components. After the installation of the equipment, the car was driven on flat and rough roads.

**Figure 4:**
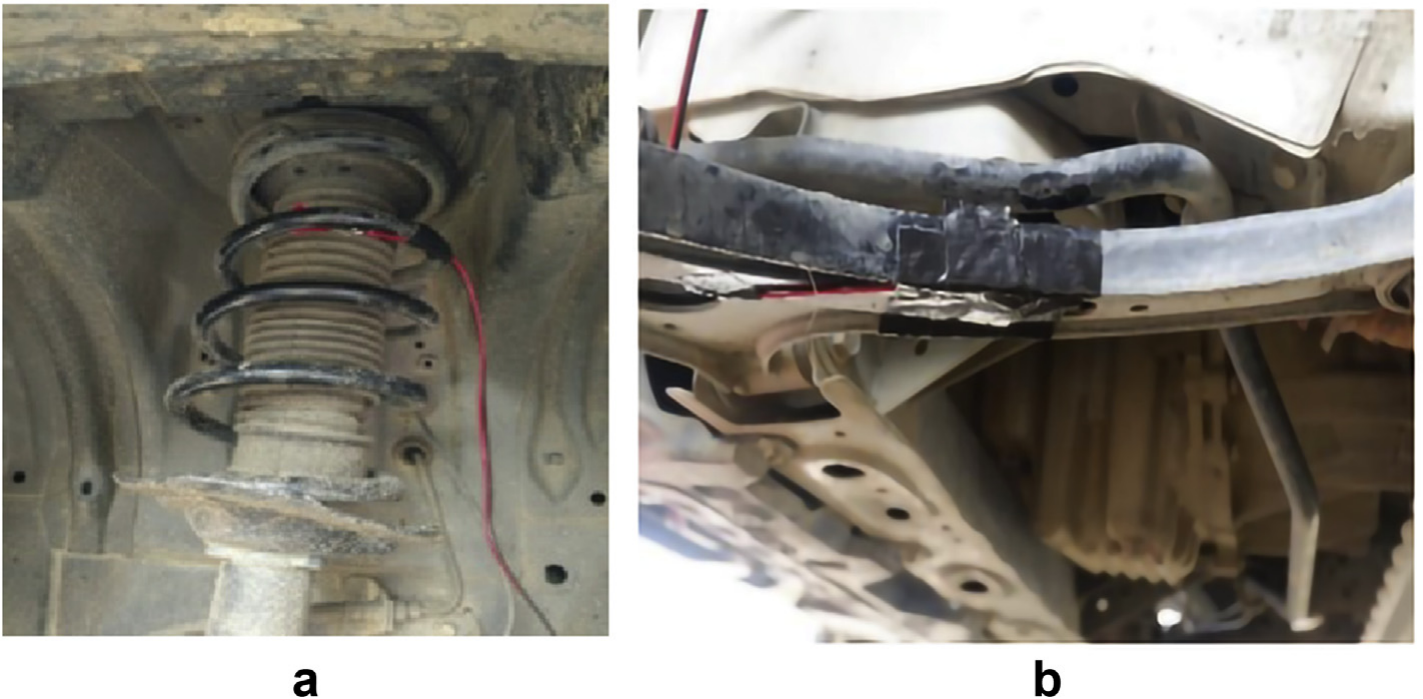
Installation of strain gauge; (a) coil spring and (b) lower arm.

**Figure 5:**
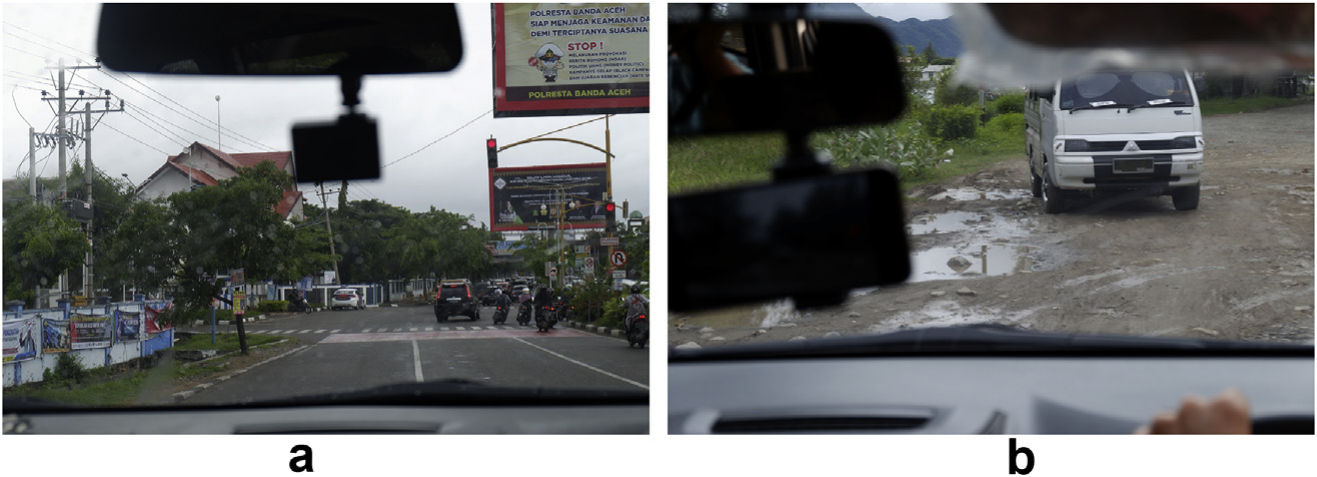
Data collection locations; (a) flat and (b) rough.

The road surface profiles were also determined by using the international roughness index (IRI) [22]. Therefore, for the road to be categorized as a flat or rough surface, the IRI value needs to be < 4 or >12, respectively. IRI can be defined as [23]:

(2) $$IRI=\frac{1}{L}\overset{T}{\underset{0}{\int }}\left|\dot{z}\right|dt$$

where $\dot{z}$ is the suspension velocity due to the road excitation, *L* is the travel distance, and *T* is the time. The car was driven at a speed over 30 km/h on the flat road and below 20 km/h on the rough road. Figure 4 shows how to install the strain gauges, and Figure 5 shows the straight road surfaces passed by the car.

### 2.4 Statistical signal parameters

For a signal *F*_*j*_ with a specific number of data *n*, the mean value $\bar{x}$ is calculated by:

(3) $$\bar{x}=\frac{1}{n}\sum_{j=1}^{n}F_{j}$$

Standard deviation (SD) is used to determine how data are distributed in a sample and is also attributed to the average variability in a data set. Its value for numbers above thirty can be stated by:

(4) $$SD={\left(\frac{1}{n}{\sum_{j=1}^{n}\left(F_{j}-\bar{x}\right)}^{2}\right)}^{1/2}$$

The root-mean square (RMS) helps to estimate the total amount of energy in discrete data. This is expressed by:

(5) $$r.m.s.={\left(\frac{1}{n}\sum_{j=1}^{n}F_{j}^{2}\right)}^{1/2}$$

Kurtosis *K* is a statistical parameter sensitive to spikes and it is estimated in discrete data using:

(6) $$K=\left(n^{-1}SD^{-4}\right){\sum_{j=1}^{n}\left(F_{j}-\bar{x}\right)}^{4}$$

### 2.5 Fatigue life assessments

Fatigue life is liable to occur in finite or infinite period, and is determined by the number of cycles. Therefore, the main point in analyzing a component subjected to repeated loading is determining the number of cycles required for fatigue failure. The most commonly utilized cycle counting method is the rain flow counting developed by Matsuishi & Endo [24]. To apply this for the whole time history, the load needs to be rearranged from the maximum peak or the minimum valley, whichever is greater in absolute magnitude. The method's main idea is to treat small cycles as interruptions to larger cycles. Small cycles are extracted at the beginning of the process, leaving larger cycles to be extracted at the end.

The fatigue life assessments were conducted based on the strain-life approach [25] such as the Coffin-Manson model [26, 27] which can be expressed by:

(7) $$\varepsilon =\frac{\sigma_{f}^{\prime }}{E}{\left(2N_{f}\right)}^{b}+\varepsilon_{f}^{\prime }{\left(2N_{f}\right)}^{c}$$

where εε is the strain amplitude, $\sigma_{f}^{\prime }$ is the fatigue strength coefficient, *E* is the material modulus of elasticity, *N*_*f*_ is the number of cycles to failure for a particular stress range and mean, *b* is the fatigue strength exponent, $\varepsilon_{f}^{\prime }$ is the fatigue ductility coefficient and *c* is the fatigue ductility exponent.

The Coffin-Manson model, however, sets the fatigue life assessment for strain loads with zero-mean stress to show the drawbacks of the relationship between the fatigue life and the strain. Therefore, two more models, Morrow [28] and Smith-Watson-Topper (SWT) [29], consider the mean stress effect [30, 31] expressed respectively by:

(8) $$\varepsilon =\frac{\sigma_{f}^{\prime }-\sigma_{mean}}{E}{\left(2N_{f}\right)}^{b}+\varepsilon_{f}^{\prime }{\left(2N_{f}\right)}^{c}$$

(9) $$\sigma_{max}\varepsilon =\frac{{\sigma_{f}^{\prime }}^{2}}{E}{\left(2N_{f}\right)}^{2b}+\sigma_{f}^{\prime }\varepsilon_{f}^{\prime }{\left(2N_{f}\right)}^{b+c}$$

where $\sigma_{\text{mean}}$ is the normal mean stress and $\sigma_{\max}$ is the maximum stress.

Fatigue damage for each loading cycle *D*_*i*_ is:

(10) $$D_{i}=N_{f}^{-1}$$

**Figure 6:**
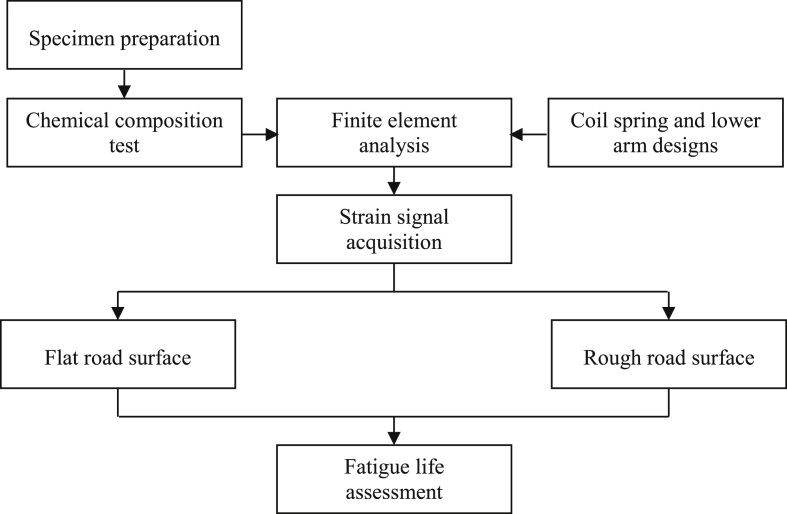
Schematic diagram of the study.

Palmgren-Miner rule [32, 33] was utilized to determine the cumulative fatigue damage, as follows:

(11) $$D=\sum \left(\frac{n_{i}}{N_{f}}\right)$$

where *n*_*i*_ is the number of applied cycles. Fatigue damage has a range of zero to one, where zero indicates no damage (infinite cycles to failure) and one is assumed as failure (one cycle to failure). The process flow of this study is summarized in Figure 6.

## 3 Results and discussion

### 3.1 Microstructural characterization

Table 1 describes the coil spring and lower arm's chemical composition. It was noted that carbon increased the strength and hardness of the components, silicon stabilized the microstructures during the tempering and the functioning operations for improving the steel's corrosion resistance, and manganese increased the ductility, wear-resistance and the components' hardness. Also, chrome and molybdenum increased the resistance to corrosion, wear-resistance, and the components' hardness, while vanadium enhanced the components' hardness. Based on the results, the coil spring was produced using the SAE 5160 carbon steel, while the lower arm was produced using the AISI 1513 carbon steel. These materials are generally used for the car suspension components' fabrication [17, 34]. The mechanical properties of the materials are shown in Table 2.

**Table 1:** The results of the chemical composition test.

Chemical elements	Coil spring		Lower arm	
	Testing results (%)	The SAE 5160 carbon steel (%) [35]	Testing results (%)	The AISI 1513 carbon steel (%) [36]
Carbon (C)	0.64	0.56–0.64	0.09	0.100–0.160
Silicon (Si)	0.16	0.15–0.3	0	0
Manganese (Mn)	0.78	0.75–1	1.33	1.10–1.40
Chrome (Cr)	0.77	0.7–0.9	0.03	0
Molybdenum (Mo)	0.18	0.15–0.25	0	0
Vanadium (V)	0.15	0.15	0.003	0

**Table 2:** The mechanical properties of the SAE 5160 and AISI 1513 carbon steel [37].

Mechanical properties	The SAE 5160 carbon steel	The AISI 1513 carbon steel
Ultimate tensile strength (MPa)	1,584	585
Modulus of elasticity (GPa)	207	210
Yield strength (MPa)	1,487	450
Fatigue strength coefficient (MPa)	2,063	1,089
Fatigue strength exponent	-0.08	-0.07
Fatigue ductility exponent	-1.05	-0.54
Fatigue ductility coefficient	9.56	0.41
Cyclic strain hardening exponent	0.05	0.11
Cyclic strength coefficient (MPa)	1,940	978
Poisson ratio	0.27	0.27

### 3.2 Stress distribution

**Figure 7:**
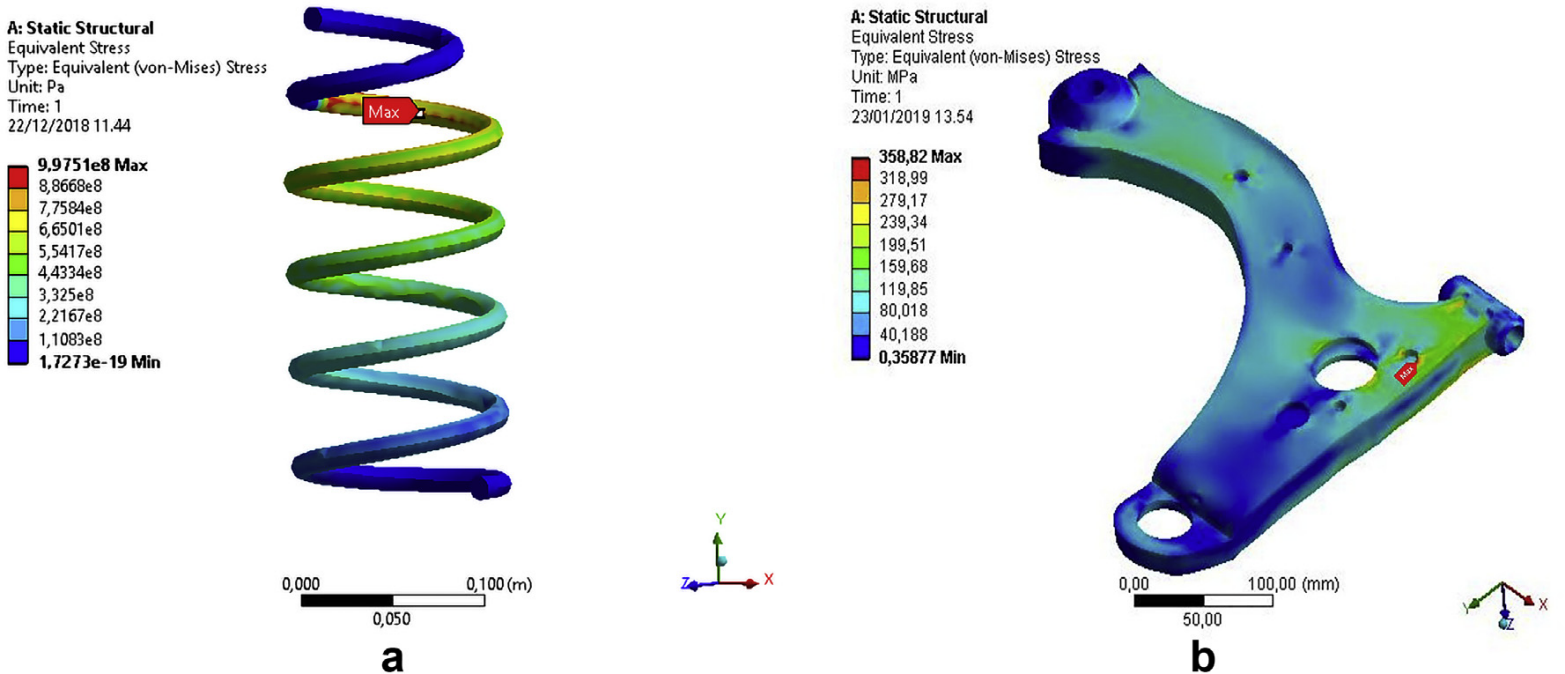
The stress distributions; (a) coil spring and (b) lower arm.

The stress distributions for the coil spring and the lower arm are available in several color contours as presented in Figure 7. The area with the highest stress concentration is denoted by red, orange, yellow, green and blue. Furthermore, the red region in the coil spring displayed the highest von Mises stress value of 997.51 MPa. According to Table 2, the maximal stress value was lesser in comparison to the SAE 5160 carbon steel's yield strength, i.e., 1,487 MPa. For the lower arm, the maximal stress value was discovered to be 358.82 MPa, which was less than the AISI 1513 carbon steel's yield strength, i.e., 450 MPa. These values indicated that the load was not responsible for these components' failure. Since the innerspring showed a lower surface area compared to the outer one, a critical point was noted on the internal spring [11, 12]. In the lower arm component's case, maximal stress was discovered in the region that was closer to the roller support at the right bushing. This was because this component contained several holes that narrowed the surface.

### 3.3 Fatigue life

**Figure 8:**
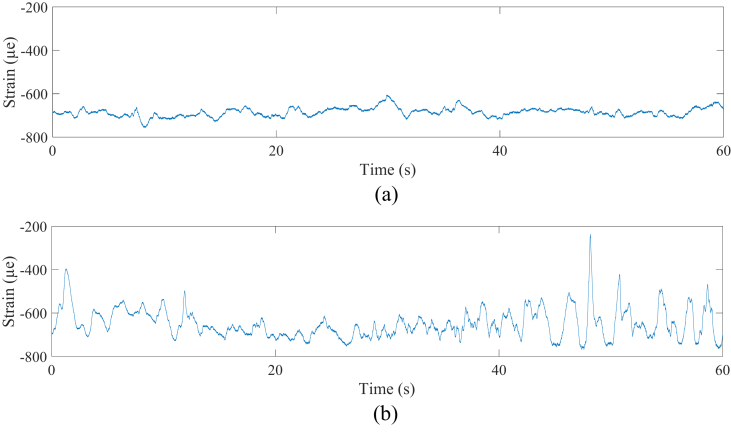
Coil spring strain signals; (a) flat (D1) and (b) rough (D2).

**Figure 9:**
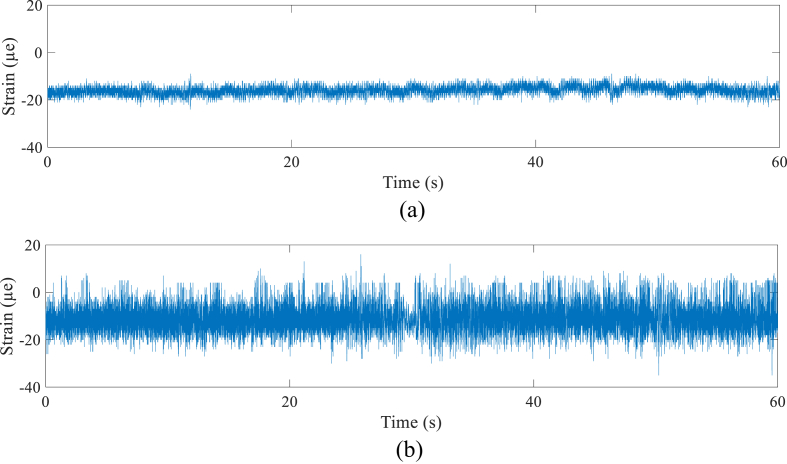
Lower arm strain signals; (a) flat (D3) and (b) rough (D4).

It was noted that the flat road surface showed an IRI value of 2.09, while the rough road surface showed 15.0. A set of 30,000 data showed a 60-seconds long strain signal. According to Figure 8, the flat road strain signal (D1) in the coil spring provided the amplitude range between -756 με and -605 με, while the rough road (D2) provided the higher amplitude range, which was between -769 με and -237 με. The flat road strain signal (D3) for the lower arm provided the amplitude range between -24 με and -9 με, while the rough road (D4) provided the higher amplitude range, which was between -35 and 16 με, as shown in Figure 9. All the strain signals had a non-zero mean value, namely -685 με for D1, -649 με for D2, -16 με for D3, and -11 με for D4, indicating the components received a compressive load.

The flat road provided the lowest strain because it had the lowest amplitude range compared to the rough road, namely 20 με for the coil spring and 2 με for the lower arm. The rough road provided the values 67 με for the coil spring and 5 με for the lower arm. However, the flat road provided higher RMS values, which were 685 με for the coil spring and 16 με for the lower arm, while the rough road provided 653 με and 12 με, respectively. The produced kurtosis value was proportional to the SD value, where both increased correspondingly. The kurtosis for each strain signal was greater than 3, indicating the strain signals could be categorized as non-stationary [38, 39].

**Figure 10:**
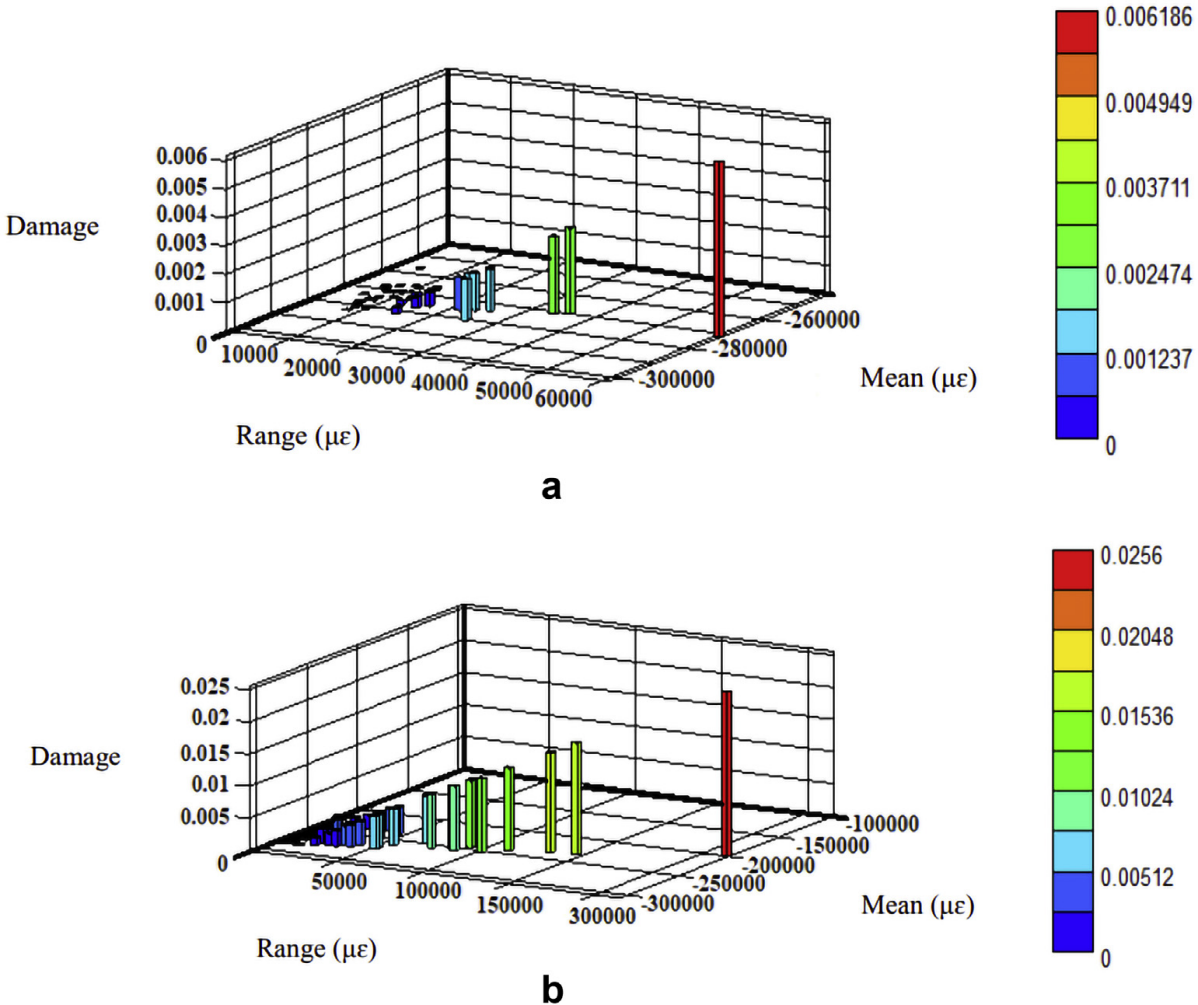
Fatigue damage on the coil spring based on the Morrow model; (a) flat and (b) rough.

**Figure 11:**
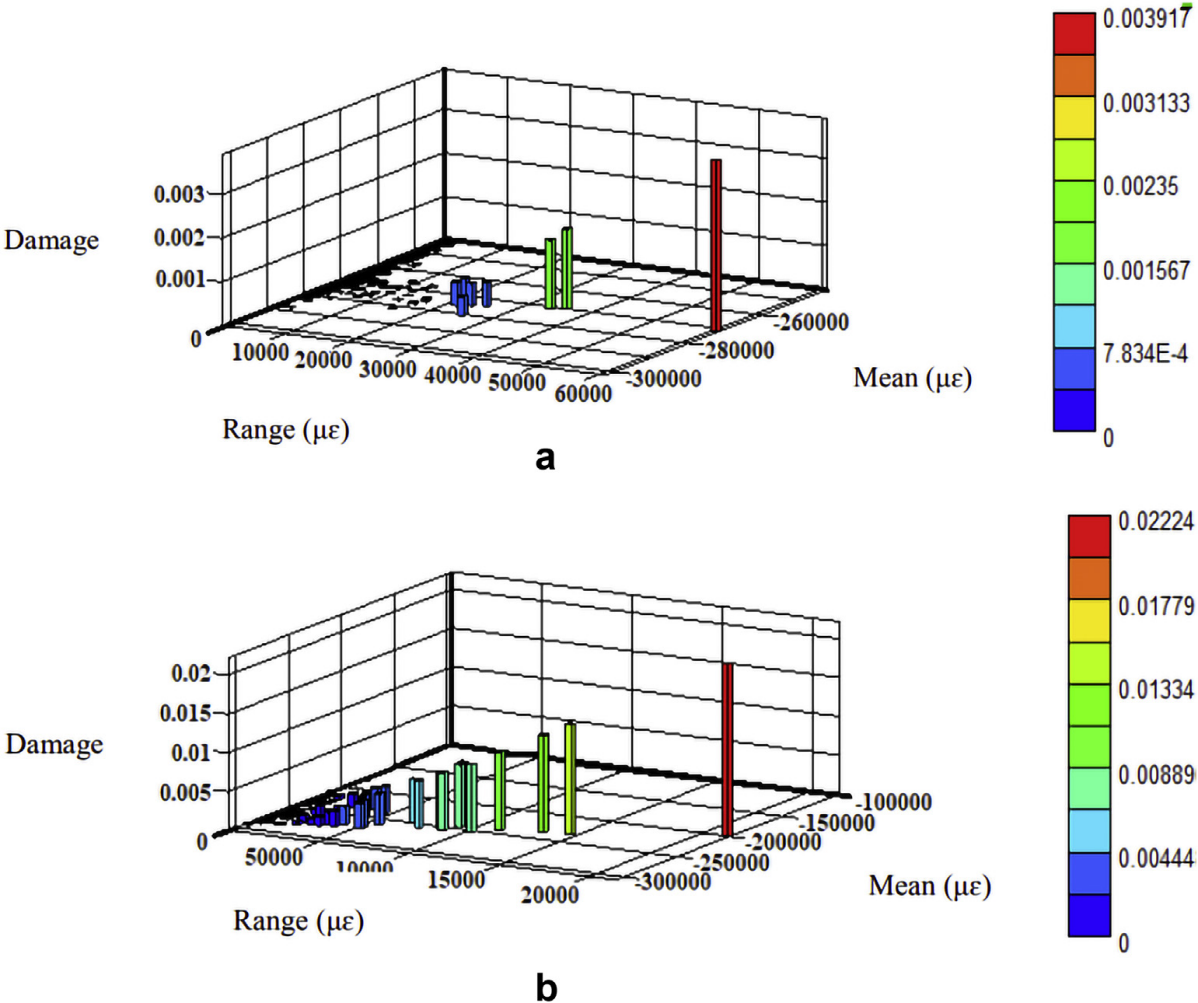
Fatigue damage on the coil spring based on the SWT model; (a) flat and (b) rough.

**Figure 12:**
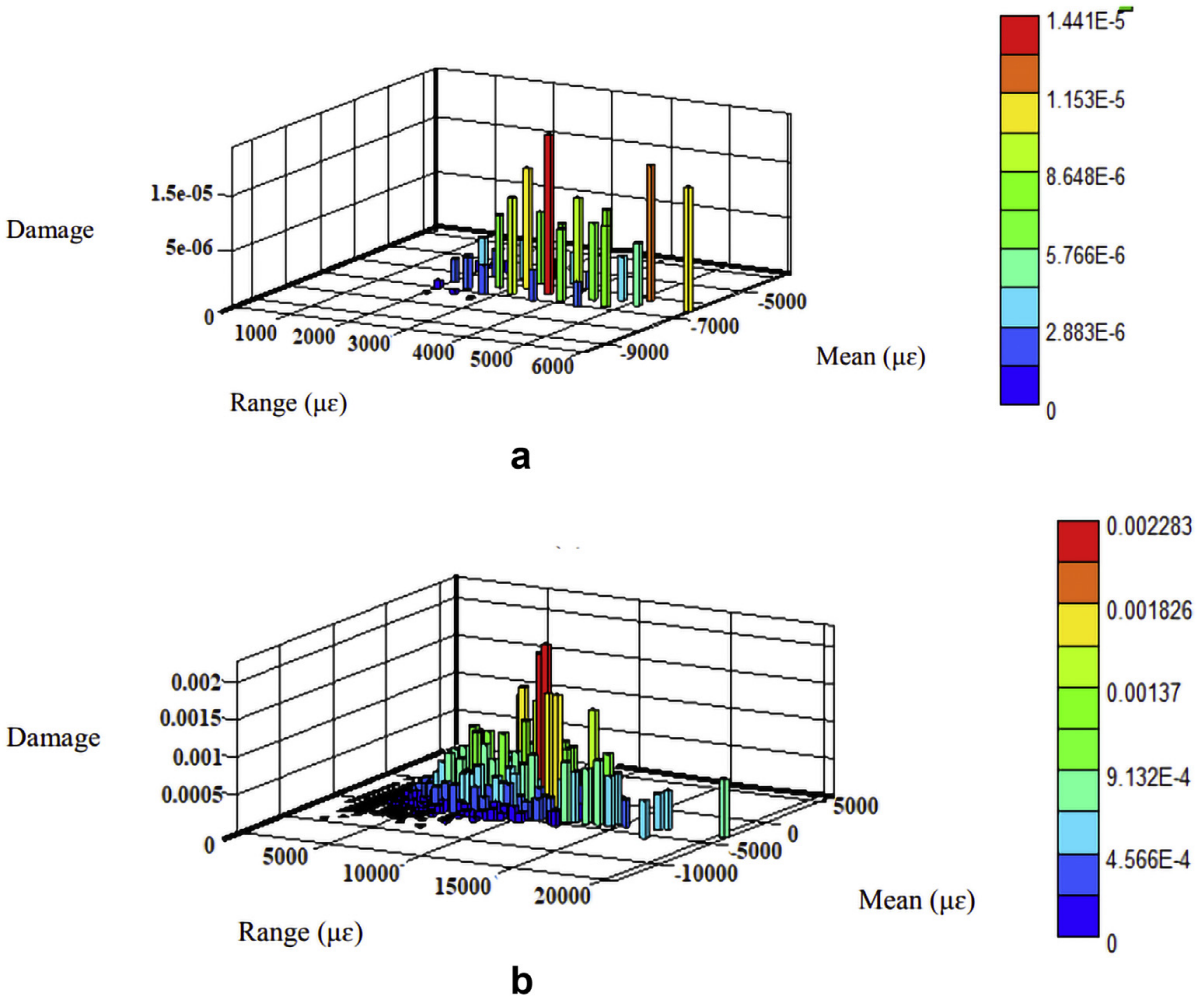
Fatigue damage on the lower arm based on the Morrow model; (a) flat and (b) rough.

**Figure 13:**
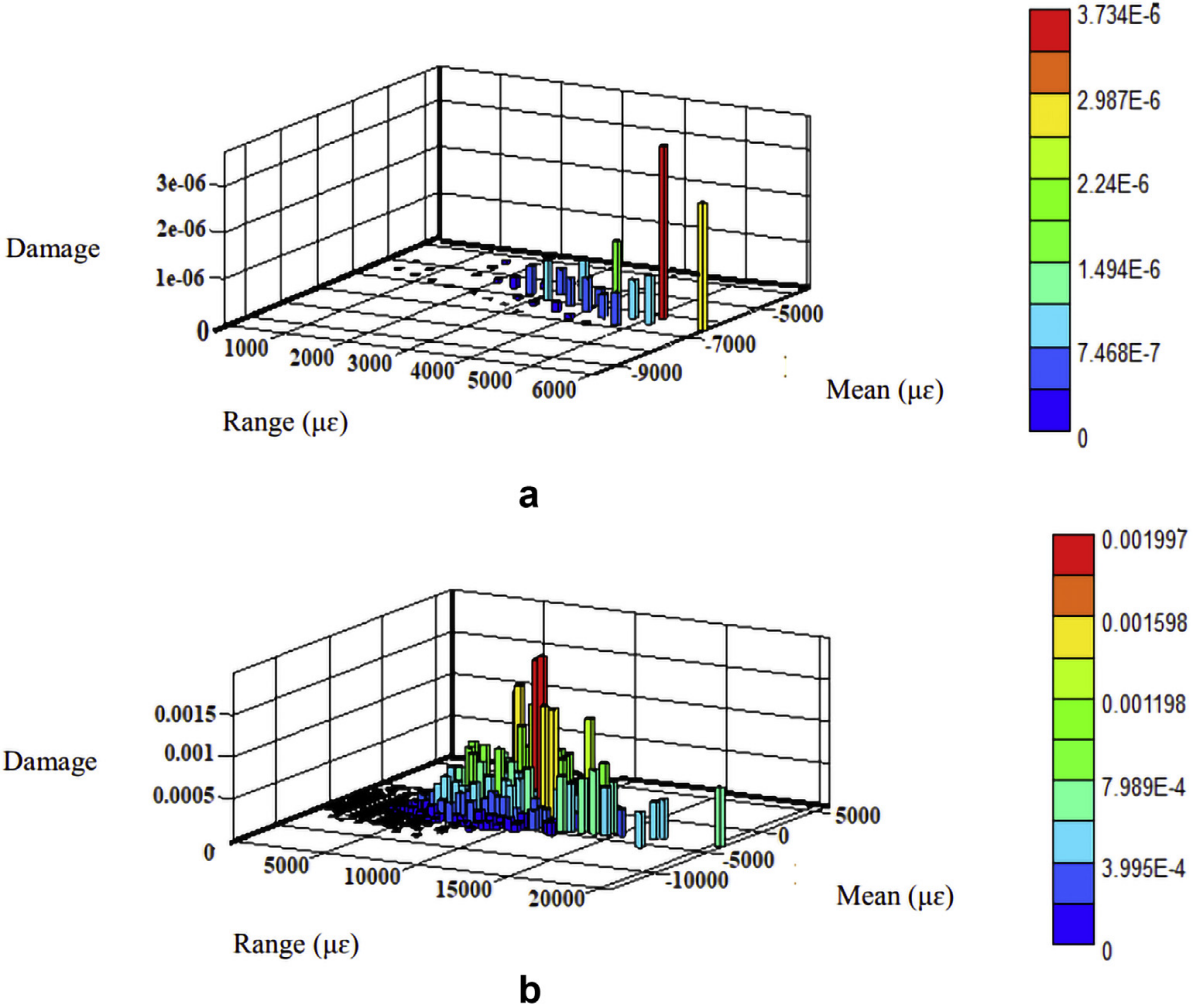
Fatigue damage on the lower arm based on the SWT model; (a) flat and (b) rough.

The fatigue damage was determined based on the Morrow and SWT models since they consider the mean stress effect. The parameters listed in Table 2 were regarded as the product of strain amplitudes to determine fatigue damage using Eqs. (8) and (9), respectively. The fatigue damage's 3-D histrograms for each model are shown in Figures 10, 11, 12, and 13. Besides, the two road surfaces showed a similar distribution pattern for fatigue damage, but with differing values. Based on the Morrow model, the fatigue damage on the coil spring for the flat and rough roads was 2.2E-02 and 2.1E-03 damage per block, respectively. For the SWT model, it was 1.0E-02 and 1.5E-01 damage per block. Meanwhile, based on the Morrow model, the fatigue damage on the lower arm for the flat and rough roads was 1.6E-04 and 9.5E-02 damage per block. For the SWT model, it was 1.7E-05 and 8.1E-02 damage per block.

The flat road had the highest fatigue life, which was 19,060 cycles to failure for the coil spring and 11,914,000 cycles to failure for the lower arm. For the rough road, the lowest fatigue life was 1,284 cycles to failure for the coil spring and 3,580 cycles to failure for the lower arm. Based on the fatigue life, the components' useful life *U*_*l*_ before failure can be predicted using the following equation:

(12) $$U_{l}=F_{l}\times S_{l}\times V$$

where *F*_*l*_ is the fatigue life, *S*_*l*_ is the signal length, and *V* is the car speed.

By inputting the fatigue life for each road, the signal length of 60 s, and the car speed of 30 km/h for the flat road and 20 km/h for the rough road, the useful life was discovered to be 9,149 km for the coil spring and 5,718,720 km for the lower arm driven on the flat road, as well as 411 km for the coil spring and 1,146 km for the lower arm driven on the rough road. According to the results, the coil spring showed almost 3 times higher failure rate compared to the lower arm when driven on a rough road, while it provided above 625 times higher failure rate when driven on a flat road. This was attributed to the fact that the road surfaces' contours imparted a vertical load, which directly affected the coil spring since it was responsible for reducing the load vertically. The coil spring played a vital role in improving ride quality. Hence, more studies need to be carried out for increasing coil springs' durability.

As reported by Putra [40], the simulated fatigue life was wide compared to the experimental fatigue life. The Palmgren-Miner rule applied to the strain-life approach depends on the assumption that sequence changes occurring in a non-uniform cycle do not affect fatigue life. Therefore, the accuracy for calculating a variable amplitude loading is doubtful. The cycle sequence contributes substantially to fatigue life, hence small cycles need not be ignored when predicting a fatigue life [41, 42, 43, 44]. The results were not fully accepted and there is a need to perform experimental fatigue tests next.

## 4 Conclusions

This study aims to identify the effect of road surface contour on coil spring and lower arm fatigue life. From the results, it was concluded that when the car accelerated on a rough road, the components received greater stresses providing a shorter fatigue life. On the rough road, the coil spring's fatigue life was 14.8 times less compared to the flat road, while that of the lower arm was almost 3,328 times less compared to the flat road. The useful life of the coil spring driven on the flat road was 9,149 km, which was more than 625 times less compared to the lower arm, while on the rough road it was only 411 km, which was almost 3 times less compared to the lower arm. The higher the fatigue damage, the lower the components' fatigue life. The coil spring failed faster than the lower arm due to the road surfaces' contour, which provided a vertical load. Hence, it experienced higher stress because the function is to reduce the load vertically, while the lower arm holds the load when turning.

## Declarations

### Author contribution statement

T.E. Putra: Conceived and designed the experiments; Wrote the paper.

Husaini: Contributed reagents, materials, analysis tools or data.

M. Ikbal: Performed the experiments; Analyzed and interpreted the data.

### Funding statement

This work was supported by 10.13039/501100015767Universitas Syiah Kuala, Indonesia (10/UN11.2.1/PT.01.03/DPRM/2021).

### Data availability statement

Data will be made available on request.

### Declaration of interests statement

The authors declare no conflict of interest.

### Additional information

No additional information is available for this paper.
